# Mind and Its Disorders: A Text-Book for Students and Practitioners

**Published:** 1909-09

**Authors:** 


**Mind and its Disorders: A Text-book for Students and Practitioners.** By W. H. Stoddart, M.D. Pp. xvi, 488. London: H. K. Lewis. 1908.

From the first issue of Griesinger's great work on mental disorders, there has proceeded a constant elaborative treatment of this subject. In its comprehensive yet perfectly homogeneous survey, the admirable character of Griesinger's treatise has never been assailed. His generalised and largely psychological treatment of the subject has been replaced of later years by works in which the results of progressive research are shown in perhaps somewhat arbitrary attempts to isolate new types and precise classifications of insanity. This we owe in great part to the continental school of investigators of more recent years, and the outcome of their efforts has been to display an almost exaggerated dissection of former broad groupings, with the object of securing more or less defined types. We think in the attempted discovery of so many entities in mental disorder that there is a risk of losing grasp of its larger and more generalised aspects, a danger of missing its important underlying principles. *Quot homines, tot sententiæ*; the variability of mental constitution and working is such, and the influence of causal and provoking factors so modifying, that symptom groupings, except of broad nature, must be unsatisfactory.

Nor is there, as yet, sufficient help from pathological findings to effect a differential classification of intimate kind. So far as the specialist is concerned, these careful partitions are of use in the forming of prognosis and influencing of treatment, but for the ordinary student and practitioner we maintain that the indication of the general characteristics of the main groupings of mental disorder, their several gravities, modifications by casual vices, by peculiar inherited conditions, states of physical health, epochs of life and so forth are of more importance. Even the asylum physician finds himself puzzled by the intricacies of diagnosis where symptoms are constantly overlapping, and affected by inexplicable minutiæ of brain organisation. How shall the student or practitioner emerge from the struggle to comprehend that amazing syndrome, so-called dementia precox? The work under notice, being a text-book, must necessarily embrace and discuss all recognised types and theories of insanity. Wc eannot, however, feel sure that the ordinary practitioner and student will undertake to assimilate the mass of information found here, and its presentation in scrupulous detail. As a book for the clinical assistant and asylum medical officer it is to be highly commended.

The work is divided into three sections. The first deals in a clear and straightforward manner with the correlation of normal mental processes and physical concomitants; the second treats similarly of morbid psychology; the last of the clinical side of insanity, with especial attention given to the neurological significance of symptoms and existing physical signs. The book is notably complete, no phase of insanity has escaped the author, and the pathological segments are brought up to date as much as is possible where most theories are still conflicting or obscure. The style is terse, and that of the clinical lecturer; illustrative instances abound, and evidently great pains have been taken to give the reader a clear and explicit portrayal of the diverse forms of mental disorder.

The details of treatment are concise and practical, and due emphasis is given to the importance of rest in bed, prolonged bath, and especially abstinence from the old-time irritating policy of "rousing" the patient in the earlier stages of his malady. The reader will note as passages of special interest the author's remarks on negative hallucinations (p. 116), a subject on which Forel and others have bestowed attention; the indicated function of the cortico-rubral system (p. 128); the relative frequency of the melancholic form of general paralysis (p. 273); the hypothetical intraneuronal intoxication or presence of irritating or paralysing products in the cortical neurons in mania and melancholia respectively (p. 215); the pathogenesis of delusion (p. 217).

The section dealing with general paralysis is especially good and complete, and anyone who wishes to gain a clear idea of what is implied by dementia precox, we think, will find in Dr. Stoddart's presentment of it the most coherent and concise account yet given. We should like, by the way, to see this term replaced by a less objectionable English one, viz. "Premature Mental Decay," which we consider does not indicate a dementia as the main feature of the process. As the significance of "dementia" in

German and English is not precisely similar, it is preferable to find a synonym in the latter tongue, rather than convey what will continue for long to be an erroneous interpretation of the disorder.

Dr. Stoddart candidly confesses that the present careful preservation and liberation of the insane procures for us the survival of the unfittest, and that the physician specialising in mental diseases is no friend to posterity. This is but true, and how far we are subject to the fetichism of conservation of merely vegetative existence is evidenced by the author on page 271, in his statement that it is his habit to tube-feed even a dying general paralytic.

The book is most creditably produced.

